# The genetic heterogeneity of colorectal cancer predisposition - guidelines for gene discovery

**DOI:** 10.1007/s13402-016-0284-6

**Published:** 2016-06-09

**Authors:** M. M. Hahn, R. M. de Voer, N. Hoogerbrugge, M. J. L. Ligtenberg, R. P. Kuiper, A. Geurts van Kessel

**Affiliations:** Department of Human Genetics, Radboud Institute of Molecular Life Sciences, Radboud University Medical Center, PO Box 9101, 6500 HB Nijmegen, The Netherlands

**Keywords:** Colorectal cancer, Genetic predisposition, Next generation sequencing, Candidate gene identification

## Abstract

**Background:**

Colorectal cancer (CRC) is a cumulative term applied to a clinically and genetically heterogeneous group of neoplasms that occur in the bowel. Based on twin studies, up to 45 % of the CRC cases may involve a heritable component. Yet, only in 5–10 % of these cases high-penetrant germline mutations are found (e.g. mutations in *APC* and DNA mismatch repair genes) that result in a familial aggregation and/or an early onset of the disease. Genome-wide association studies have revealed that another ~5 % of the CRC cases may be explained by a cumulative effect of low-penetrant risk factors. Recent attempts to identify novel genetic factors using whole exome and whole genome sequencing has proven to be difficult since the remaining, yet to be discovered, high penetrant CRC predisposing genes appear to be rare. In addition, most of the moderately penetrant candidate genes identified so far have not been confirmed in independent cohorts. Based on literature examples, we here discuss how careful patient and cohort selection, candidate gene and variant selection, and corroborative evidence may be employed to facilitate the discovery of novel CRC predisposing genes.

**Conclusions:**

The picture emerges that the genetic predisposition to CRC is heterogeneous, involving complex interplays between common and rare (inter)genic variants with different penetrances. It is anticipated, however, that the use of large clinically well-defined patient and control datasets, together with improved functional and technical possibilities, will yield enough power to unravel this complex interplay and to generate accurate individualized estimates for the risk to develop CRC.

## Colorectal cancer

Colorectal cancer (CRC) is the third most commonly diagnosed cancer in males and the second in females, with worldwide over 1.3 million new cancer cases and over 650,000 deaths reported each year [1]. The incidence of CRC is strongly age-dependent, increasing from the age of 40 years and reaching a median age of 70 years in the general population [2]. CRC is not a single disease, but rather a heterogeneous group of malignancies originating from precursor cells within the gastrointestinal tract between the cecum and the anus [3]. Clinically, these malignancies can differ in (i) localization (e.g. proximal or distal), (ii) pathology/histology (e.g. adenocarcinoma/serrated adenocarcinoma) and (iii) invasiveness/metastatic behaviour (e.g. loco-regional or distant organ site) [3]. About 90 % of CRCs present as adenocarcinomas originating from the epithelial lining of the colon [4]. Other types of CRC include neuroendocrine, adenosquamous, signet ring cell, squamous cell, spindle cell and undifferentiated carcinomas [4]. Although every CRC is unique, it is generally believed that tumours with similar clinicopathologic characteristics arise and behave in a similar way [5]. Along with the increase in our understanding of the pathology and etiology of CRCs in recent decades, improved classification systems have been developed [5, 6]. An example of this progress is the development of the pathological Dukes classification system [7] to the TNM staging system that grades tumour growth and dissemination to lymph nodes and other organ sites [8]. Recently, high-resolution molecular profiling of somatic mutations has resulted in new insights in CRC sub-phenotyping [9]. While the complete mutation profiles are, as yet, complex and not well enough understood to be used in routine clinical practice, classification systems have been introduced that primarily rely on specific mutation spectra [6].

## Etiologic and pathologic hallmarks of CRC

A milestone in the classification of CRCs was achieved when it was first postulated in the late 1980’s that CRCs develop via a multistep process that is accompanied by the sequential accumulation of genetic mutations, often occurring over many years [10]. A more detailed comprehension of this multistep processes may provide a basis for understanding how various developmental and pathological characteristics of CRCs contribute to the apparent heterogeneous manifestation of this disease.

The first formulation of the multistep carcinogenic process was postulated by Fearon and Vogelstein, who described the timing of genetic mutations that induce chromosomal instability (CIN) [11]. The CIN pathway includes molecular events that occur during the development from hyperproliferative colon epithelium to adenomatous lesions and, finally, invasive adenocarcinoma. It has been put forward that mutations in so-called ‘gatekeeper genes’ are the major drivers of tumorigenesis [12]. Based on a genetic study of early adenomatous lesions it was found that the majority of the earliest lesions carry a mutated, inactive, *APC* gene [13]. Concurrently, it was found that the frequency of inactivating *APC* mutations remained constant as tumours progressed from benign to malignant stages [13]. Based on these findings, inactivation of *APC* was proposed to represent an initiating event of the CIN pathway. Another genetic event conferring neoplastic properties to colonic cells was found to occur in the *KRAS* proto-oncogene in intermediate adenomas [10, 11]. Currently, it is thought that additional mutations that further propagate or ‘drive’ tumour development target genes and/or pathways that lead to various developmental and pathological hallmarks of cancer. These hallmarks include, for instance, the evasion from growth control (TGF-β signalling [14]), the acquisition of chromosomal/mitotic instability (spindle-assembly checkpoint [15, 16]) and the loss of cell cycle control and apoptosis (p53 pathway [17]). As a consequence, mutations that are required for CRC development can affect any of the pathways mentioned, which hence may lead to molecular and genetic heterogeneity [18]. In addition to the CIN pathway, at least two alternative pathways have been proposed, i.e., the CpG island methylator phenotype (CIMP) pathway and the microsatellite instability (MSI) pathway.

The CIMP and MSI pathways are characterized by different mutational mechanisms, but they also lead to the acquisition of cancer-specific features reminiscent to those seen in the CIN pathway [18]. The CIMP phenotype is related to widespread promoter CpG island methylation [5, 6]. Tumours with a CIMP ‘high’ phenotype are thought to arise from sessile serrated adenomas and these tumours carry specific mutations in the *BRAF* gene reminiscent to those encountered in the *KRAS* gene in CIN tumours [18]. It has been suggested that the CpG island methylation observed may silence specific tumour-related genes, including the mismatch repair gene *MLH1*, which may give rise to microsatellite instable tumours [5, 6]. MSI-positive tumours tend to have stable karyotypes (CIN-negative). Instead, their genomes feature instability at e.g. mono- or dinucleotide repeats such as (A)n or (CA)n, referred to as microsatellites [19]. The MSI phenotype can also manifest itself independent of CIMP. It has been shown that biallelic somatic mutations in the *MLH1* or *MSH2* genes can explain a sizeable fraction of tumours that are characterized by microsatellite instability [20–22]. These tumours tend to feature *KRAS* mutations more frequently than CIMP tumours [5, 6]. It should be noted, however, that the CIN, CIMP and MSI pathways are non-exclusive, and that alternative pathways and clinical entities are still being discovered as well [23–29]. In addition to somatic mutations, also, germline mutations may affect the ultimate tumour characteristics.

## CRC predisposition syndromes

Concurrent with the formulation of the adenoma-carcinoma sequence in sporadic tumours, fine mapping of genomic lesions in the germline of patients with familial adenomatous polyposis (FAP) syndrome has pointed at the same *APC* gene [30–32]. It is assumed that in these patients germline inactivation of one *APC* allele markedly increases the chance of adenoma formation and that somatic inactivation of the second *APC* allele is a critical (rate-limiting) event in adenoma formation [18]. Presumably, once adenomas in FAP have developed, they progress at a rate akin to that seen in sporadic adenomas because of the need to acquire additional rate-limiting mutations for carcinoma development [18].

Subsequently, the genes underlying the development of a second distinct group of familial tumours, now known as Lynch Syndrome, were identified. Patients with this syndrome differ from FAP patients in that they generally show a non-polyposis phenotype, with CRCs at a relatively young age and an excess of extra-colonic tumours [33]. The genes underlying Lynch Syndrome that were consecutively identified include *MSH2* [34, 35], *MLH1* [36, 37], *PMS2* [38] and *MSH6* [39]. In addition, our group found that 3′ *EPCAM* deletions underlie a heritable form of epigenetic silencing of the *MSH2* gene [40]. It has been proposed that Lynch tumours result from major increases in mutation rates in the adenomatous lesions itself, thus leading to an accelerated progression towards carcinomas [18].

To facilitate the identification of individuals at risk for CRC development, clinical guidelines have been issued [41], including (i) a strong family history of colorectal cancers or polyps, (ii) multiple primary cancers in a patient with CRC, (iii) the occurrence of other cancers within a kindred consistent with a known CRC causing syndrome, and (iv) a relatively young age at the initial time of diagnosis. For a suspicion of specific syndromes further criteria may be added. For instance, FAP will generally be suspected in patients when at least 100 colonic adenomas are identified [42], and the presence of microsatellite instability (MSI) in the tumour in combination with a positive family history may serve as hallmarks of Lynch syndrome [43]. Besides its specific application to identify patients for example in FAP and Lynch syndrome, such clinical guidelines have also been used to distinguish additional CRC predisposition syndromes from sporadic forms of CRC.

## Additional CRC predisposition syndromes

In addition to the most prevalent FAP and Lynch syndromes, germline mutations and aberrations in pathways relevant to cancer development have been identified in more rare highly penetrant Mendelian CRC predisposition syndromes (Table 1). Many of these genes were identified through targeted investigation of candidate genes. Among the first syndromes to be discovered this way were the hamartomatous polyposis syndromes Peutz-Jegher’s syndrome (PJS) and juvenile polyposis syndrome (JPS). These syndromes are characterized by mutations in the chromatin remodelling gene *LBK1*/*STK11* [44, 45] and the TGF-β signalling genes *SMAD4* and *BMPR1A* [46, 47], respectively. In later studies autosomal recessively inherited mutations in the DNA repair gene *MUTYH* were found to give rise to a specific subtype of polyposis, referred to as *MUTYH*-associated polyposis (MAP) [23]. Recently, genome-wide screening efforts led to the identification of mutations affecting the expression of *GREM1* as the cause of hereditary mixed polyposis syndrome (HMPS) [48] and mutations in the *POLD1* and *POLE* genes as the cause of polymerase proofreading-associated polyposis (PPAP) syndrome [27]. In addition, our group very recently identified mutations in the *NTHL1* gene as the cause of *NTHL1*-associated polyposis (NAP) syndrome [29].

**Table 1:** Known CRC predisposition syndromes and CRC-associated cancer syndromes, including the genes and pathways involved

Syndromes	Genes involved	Pathways involved
Mendelian colorectal cancer syndromes		
Familial adenomatous polyposis syndrome	*APC*	WNT-signalling cascade
Lynch syndrome	*MSH2*, *EPCAM*, *MLH1*, *MSH6*, *PMS2*	Mismatch repair cascade
Peutz-Jegher’s syndrome	*STK11*	Chromatin remodelling cascade
Juvenile polyposis syndrome	*SMAD4*, *BMPR1A*	TGFβ-signalling cascade
Polymerase proofreading associated polyposis syndrome	*POLD1*, *POLE*	Polymerase proofreading cascade
MUTYH-associated polyposis syndrome	*MUTYH*	Base-excision repair cascade
NTHL1- associated polyposis syndrome	*NTHL1*	Base-excision repair cascade
Other Mendelian syndromes associated with CRC		
Bloom syndrome	*BLM*	RecQ-DNA helicase cascade
Cowden syndrome	*PTEN*	AKT-signalling cascade
Oligodontia-colorectal cancer syndrome	*AXIN2*	WNT-signalling cascade
Li-Fraumeni syndrome	*TP53*	Cell-cycle control cascade

Besides the above-mentioned Mendelian CRC syndromes, CRC predisposition has also been observed in patients affected by various developmental syndromes (Table 1), including Bloom syndrome, which is caused by mutations in the *BLM* gene [49]. Also oligodontia-colorectal cancer syndrome, which is caused by defects in the *AXIN2* gene, is associated with CRC predisposition [50]. In addition, an increased CRC risk has been reported in cases of Cowden syndrome [51] and Li-Fraumeni syndrome [52], which are caused by defects in the AKT signalling regulator *PTEN* and the cell cycle regulator *TP53*, respectively.

## Missing heritability of CRC

**Fig. 1:**
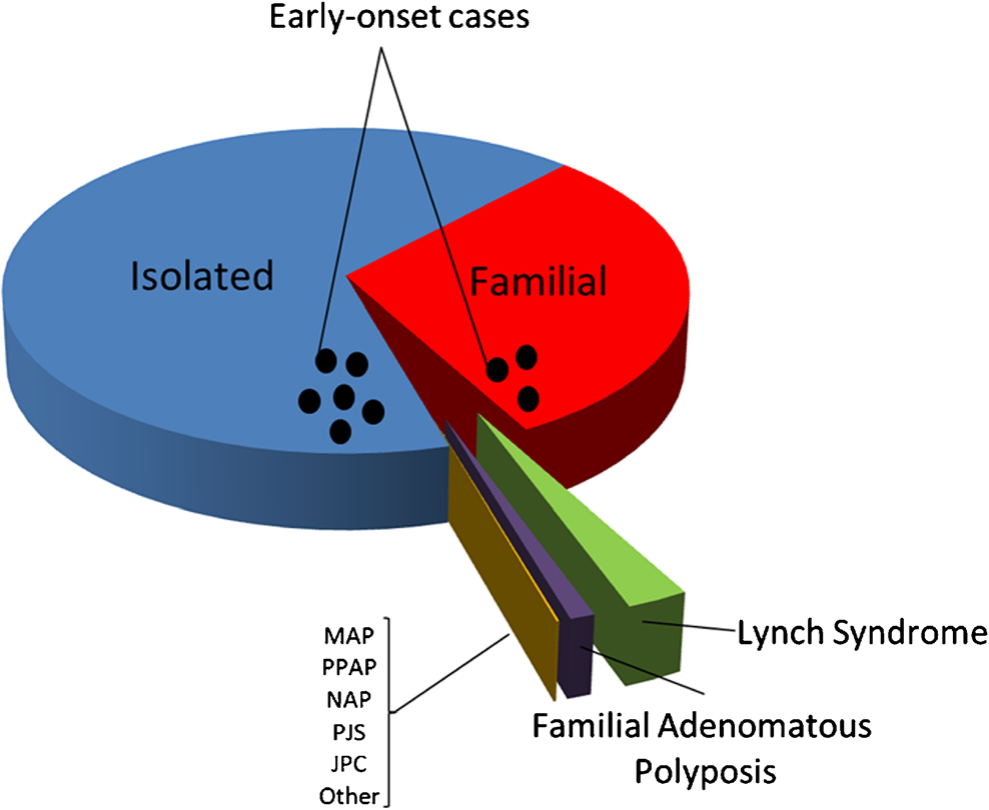
Distribution of CRC cases by familial background. Colorectal cancers can be divided into isolated cases, familial cases and hereditary cases. The latter result from known predisposition syndromes, like Lynch syndrome and familial adenomatous polyposis (FAP) syndrome. Additional, less frequent syndromes include polymerase proofreading-associated polyposis (PPAP), MUTYH-associated-polyposis (MAP), Peutz-Jegher’s syndrome (PJS), Juvenile Polyposis syndrome (JPS), NTHL1-associated polyposis (NAP), and some other colorectal cancer-associated syndromes as described in the main text. Currently, these forms of CRC predisposition account for 5–10 % of all cases. The black dots represent additional early-onset cases below the age of 50. About 10 % of CRC cases belong to this group. These cases can thus be familial or sporadic in origin and may result from variability in penetrance of the underlying risk alleles as described in the text. Modified from [53, 54].

Together, the above-mentioned Mendelian cancer syndromes account for ~5–10 % of the total burden of CRC [33] (Fig. 1). Based on twin studies from the Swedish, Danish and Finnish twin registries, the heritability of CRC was suggested to be on average ~ 30 % (range: 15–45 %) [55–57]. The apparent discrepancy between the fraction accounted for by Mendelian cancer syndromes and the aforementioned estimates have led to the notion of ‘missing heritability’ [58–60]. It is thought that this missing heritability may be multifactorial in nature, i.e., may be associated with moderately to lowly penetrant genetic variants that, possibly in conjunction with environmental factors, may fail to give rise to clear-cut dominant or recessive inheritance patterns [61]. The more common heritable forms may still include some rare high-risk Mendelian non-syndromic forms of CRC, but likely exist predominantly of intermediate familial risk forms of CRC and relatively low-risk forms of CRC that depend on interactions between genetic and environmental factors [61, 62] (Fig. 1).

In the search for this missing heritability, two contrasting hypotheses have been put forward: the common disease-common variant (CDCV) hypothesis and the common disease-rare variant (CDRV) hypothesis [63]. The CDCV hypothesis postulates that low-risk susceptibility variants occurring in >1 % of the population may contribute to the development of CRC [63]. In contrast, the CDRV hypothesis postulates that many rare variants, with frequencies <1 % in the population, may contribute to the development of CRC [61, 63]. Most of the initial candidate gene studies lacked the power and genomic resolution to address this latter hypothesis [64] and high-throughput identification of variants found in more than 1 % of the population only became feasible after the introduction of massively parallel genotyping techniques enabling genome-wide association studies (GWAS).

## Genome-wide association studies

Since 2007, several genome-wide association studies (GWAS) have been conducted under the premise of the CDCV hypothesis for CRC [65–84]. These studies aimed to use unbiased genome-wide screens to identify non-random associations of alleles at nearby loci (linkage disequilibrium), which then served as proxies, or tagSNPs, for other nearby risk alleles [85]. To adequately power the detection of risk variants with small effect sizes through GWAS, the sizes of the case-control cohorts in the discovery phase have steadily been growing from roughly 1000 cases and controls included in the initial studies [65–67] to (meta)analyses of several 10,000 samples in the more recent studies [80, 86, 87]. In addition to these large cohorts, recent studies have also started to address the involvement of multi-ethnic disease loci [88].

Together, GWAS have led to the identification of approximately 25 distinct CRC risk loci [60, 89]. Some of the variants associated with CRC risk are in or near genes implicated in relevant functional pathways, such as the DNA repair [75], TGF-ß and MYC signalling pathways [65, 68–70, 80, 87]. As yet, however, a major fraction of the associated variants cannot be placed within any functional context [90]. Due to the small effect-sizes of the loci identified in GWAS, the clinical utility of the outcome of these studies has so far been limited [60, 89]. It has been estimated that individuals carrying multiple low-penetrance risk loci may have a 1–2 fold increase in their lifetime CRC risk [89]. Together, these common variants, including those that remain to be discovered, may explain another 5–10 % of the heritability of CRC [89], suggesting that a substantial fraction of its heritability may still be missing.

## Common disease - rare variant hypothesis

The remainder of the CRC risk may be due to rare variants causing common non-Mendelian forms of CRC (CDRV paradigm) [61]. Features reminiscent to those of Mendelian syndromes characterize these CRCs in that they may occur at relatively young ages and may be found as familial aggregates. They are, however, distinct from the above-mentioned Mendelian forms in that the CRC patients usually do not exhibit a clearly recognizable clinical phenotype. Examples of the latter are the identification of germline mutations in the *EPHB2* and *GALNT12* genes through targeted screens of familial CRC patients [91–93]. Additionally, our group has previously implicated *PTPRJ* [94], *BUB1* and *BUB3* [95] variants in familial and early-onset CRC cases without polyposis and/or loss of mismatch repair capacity, which is again indicative of a contribution of rare variants to CRC predisposition.

**Fig. 2:**
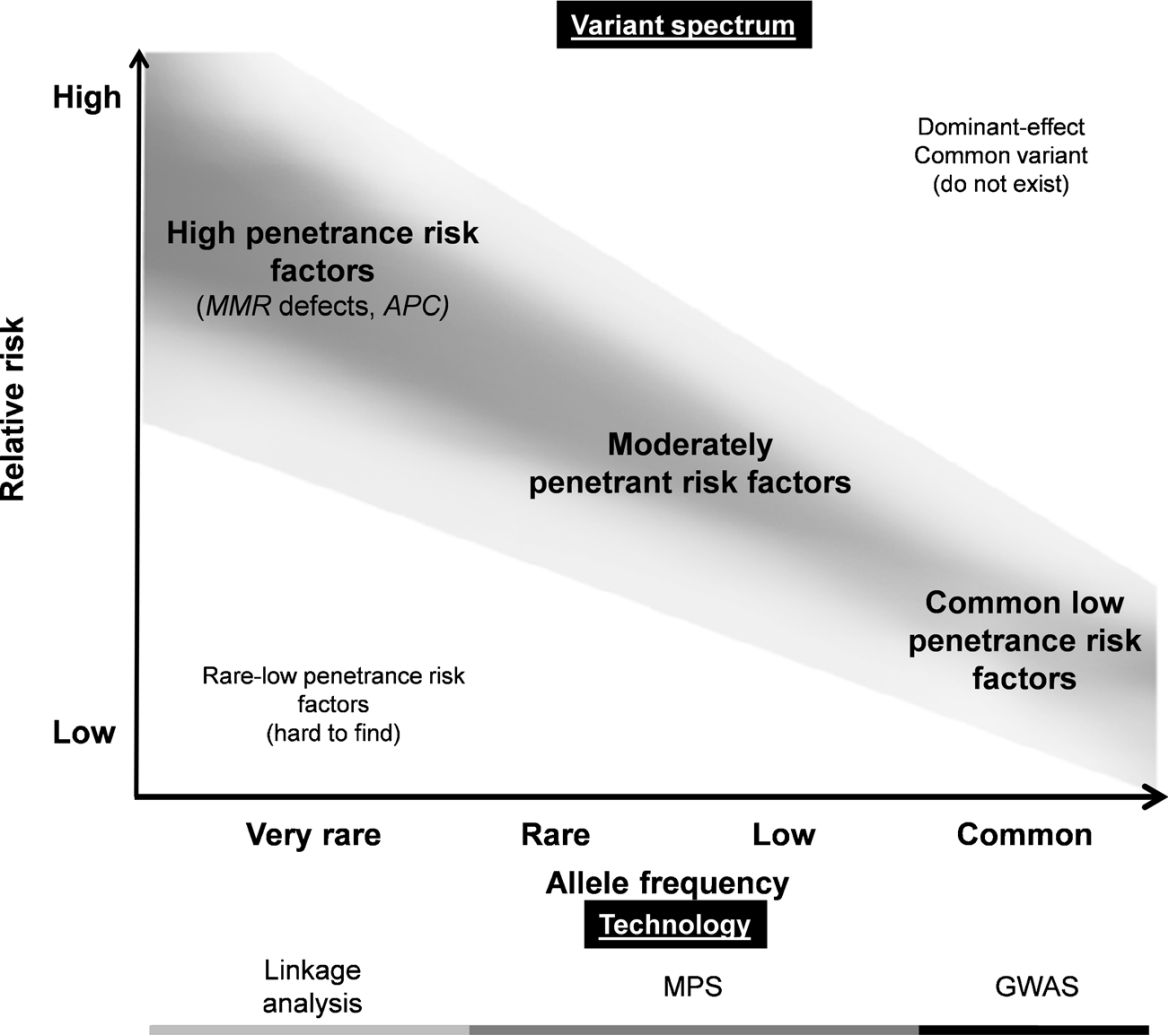
Model explaining the penetrance of genetic risk factors for CRC. In the top panel, the genetic risk factors are shown. The risk factors that, according to current knowledge, do contribute most to CRC are shown in the grey shaded bar. Highly penetrant mutations, like those found in FAP syndrome, Lynch syndrome and other hereditary/familial syndromes, are at the top left side of this plot. Usually, these syndromes are caused by very rare mutations. At the lower right side common low-penetrance risk factors are depicted. In between these two extremes, rare moderately penetrant risk factors may exist that still await discovery. The bottom panel marks the technologies that have led to the discovery of the various genetic risk factors, i.e., the light grey bar marks highly penetrant risk factors that have been identified by linkage analysis, the *black bar* marks risk factors that have been identified by GWAS. In addition, massively parallel sequencing (MPS) may allow the identification of additional moderately penetrant risk factors between these two extremes (dark grey bar)

Based on current knowledge of CRC susceptibility, a model has been suggested and expectations about the remaining genetic risk factors have been formulated (depicted in Fig. 2). According to this model, one side of the risk spectrum represents the highly penetrant variants underlying e.g. Mendelian cancer syndromes, and the other side of the risk spectrum represents the common low-penetrance variants identified by GWAS [62, 96]. It has been suggested that the majority of the missing heritability may involve variants with low minor allele frequencies (MAF), defined here as 0.5 % < MAF < 5 %, or rare variants (MAF < 0.5 %) [96]. Such variants are not frequent enough to be captured by current GWAS, nor do they exhibit sufficiently large effect sizes to be detected by classical linkage analyses in families [96]. Indeed, many of the above-mentioned examples of rare variants fall into this category [96]. Recently, the possibility has arisen to study more rare genomic variants through unbiased genome-wide screens using massively parallel sequencing (MPS) [97, 98] (Fig. 2). Indeed, recent reports implicating variants in *RPS20* [28] and *FAN1* [99] in familial CRC predisposition support the potential of MPS to identify rare variants with a moderate penetrance.

## Massively parallel sequencing and cancer predisposition

While it took almost 20 years for the first draft of the human genome to be completed [100, 101], the recent application of MPS, and in particular whole-exome and whole-genome sequencing (WES and WGS, respectively), allows the generation of comparable data within a few days at highly reduced costs [97, 102, 103]. Particularly for Mendelian traits, MPS has allowed the identification of numerous disease genes (reviewed in [104–107]) including a number of genes underlying rare highly penetrant well-phenotyped cancer predisposition syndromes. For CRC, this approach has resulted in the identification of distinct syndromes, such as PPAP [27] and NAP [29], which are caused by rare variants with a presumably high penetrance. Currently, it is unclear how many CRC cases can be explained by these syndromes, as the exact frequencies of the underlying variants still have to be established.

Next to these highly penetrant risk factors, the identification of low- to moderately-penetrant CRC risk factors remains of major interest [108]. Their identification, however, remains complex. Studies that focused on the identification of such risk factors were mainly based on (i) familial CRC cases [99, 109–111], (ii) sporadic early-onset CRC cases [112, 113] or (iii) combinations of such categories [57, 114, 115]. Unfortunately, the cohort sizes in these studies have thus far been relatively small and the identified candidates have not always been confirmed in independent studies. A good example of the latter are pathogenic mutations in the *BLM* gene. Recently, we identified two carriers of a pathogenic *BLM* mutation by exome sequencing in a cohort of 55 early-onset CRC patients (≤ 45 years of age) [115], the relevance of which could be confirmed in an additional larger cohort of early-onset CRC patients [116]. Still, the interpretation of studies aimed at the detection of low- to moderately-penetrant CRC factors remains difficult.

Several factors may underlie the complexity and limited success of these studies. First, many large-scale sequencing projects have yielded the conclusion that the human genome harbours a plethora of non-causative, possibly detrimental, rare variants [117–122]. As a result, it has become a major interpretation challenge to point out the causative variants and genes within a vast number of candidates. Secondly, these variants may not lead to obvious inheritance patterns per se, since their ultimate effect on the phenotype may rely on an interplay with other genetic and/or environmental factors [62]. Consequently, not all referral criteria used for the identification of high risk syndromes as outlined above may apply. Conversely, the distinction from individuals with sporadic non-inherited tumours is not clear-cut, making it impossible to distinguish between cases with or without predisposition on a case-by-case basis. Thirdly, since corroborative evidence is still missing for many novel candidates, and many variants and genes remain to be discovered, there is a lack of knowledge about the biological specifications of such variants.

## Guidelines for gene discovery

### Lessons learned from Mendelian cancer syndromes

In 2009, *PALB2* was reported as the first candidate cancer predisposition gene discovered by MPS [123]. Since then, MPS has been spearheading identification strategies for novel cancer predisposition genes. Especially the discovery of novel Mendelian forms of cancer predisposition from well-selected cohorts of patients by WES and WGS appeared to be successful. This notion is exemplified by studies that have led to the identification of various novel candidate cancer predisposing genes, including *GATA2*, *MAX*, *PALB2*, *SMARCE1*, *BAP1* and *ERCC4*, which predispose to the clinically well-defined entities Emberger-syndrome [124], pheochromocytoma [125], multiple spinal meningioma [126], melanocytic tumours [127] and Fanconi anaemia [128], respectively.

**Fig. 3:**
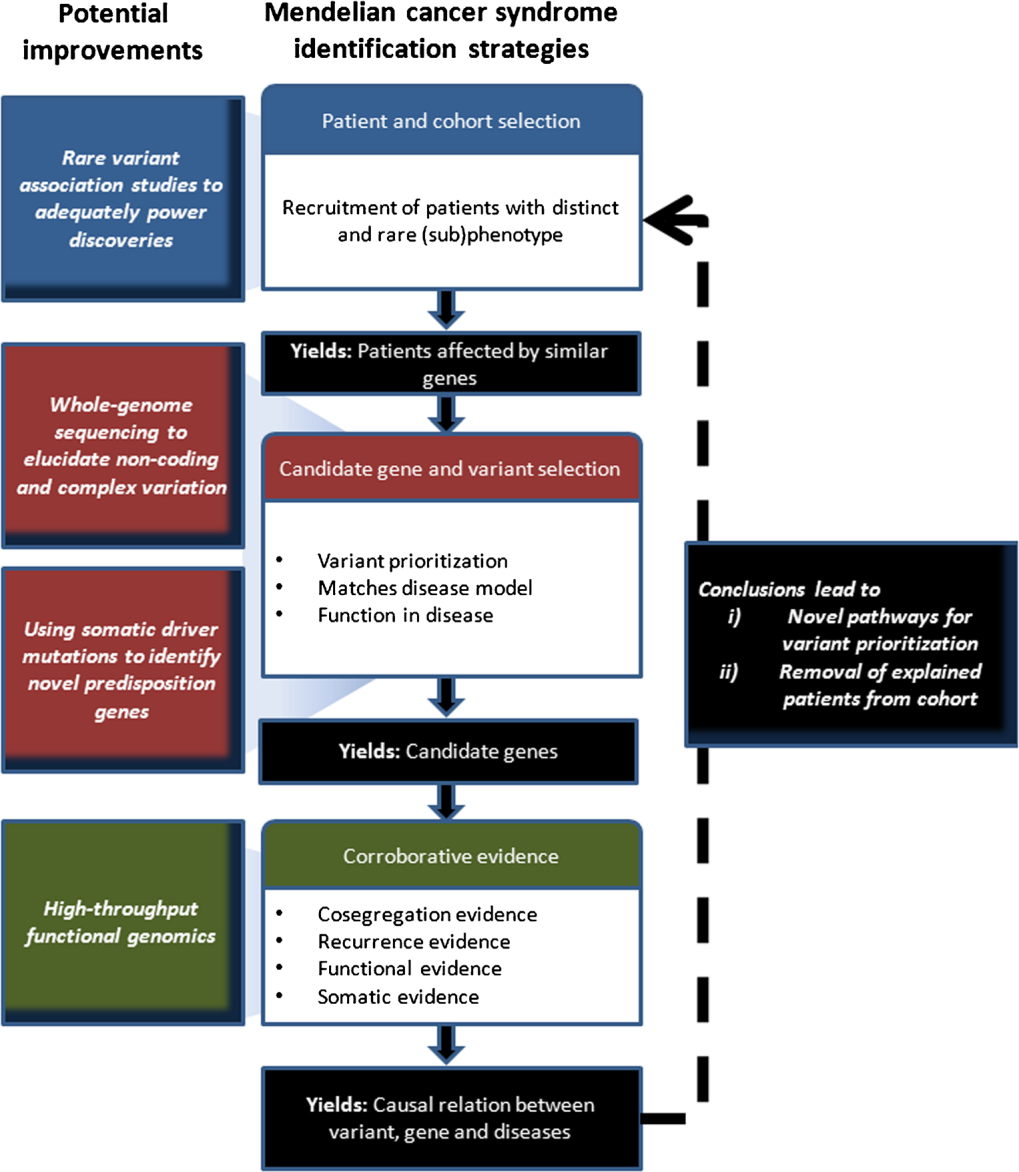
Strategy for (colorectal) cancer predisposition gene identification. Commonalities of recently published successful MPS studies to identify cancer predisposition genes in hereditary CRC and potential improvements as described in the main text

Beyond the successful identification of novel candidate genes for the rare cancer syndromes mentioned above, the methodologies and guidelines applied to these syndromes have benefited largely from those used in syndromes with Mendelian inheritance patterns [106, 107, 129]. As such, the success rates appear to be similar to the ~0.5 genes identified per disease studied with a Mendelian inheritance pattern [106]. The methodologies and criteria used to study these diseases encompass a couple of distinct entities, i.e., (1) patient and cohort selection, (2) candidate gene and variant selection and (3) corroborative evidence to support causality, including co-segregation, recurrence, functional and somatic evidence (Fig. 3).

#### Patient and control cohort selection

A well-designed study starts with a carefully selected cohort of patients and/or families, which will increase the chance to discover rare disease-causing candidate genes and/or variants (Fig. 3). Unfortunately, however, it is not always possible to select well-phenotyped cases. For some cancer syndromes, which are genetically homogeneous, this may not be a problem since candidate variants will be implicated through frequency if the number of cases included is large enough. This has recently been demonstrated e.g. by the finding of germline *CTR9* mutations in 3 out of 35 patients with Wilms tumour [130]. However, for conditions such as CRC, the current cohorts of early-onset and familial cases appear to be too heterogeneous to use such strategies. It may, therefore, be necessary to apply more stringent definitions by e.g. performing endophenotyping (see below) of tumours or by focusing on rare phenotypes such as the occurrence of childhood CRC. In this way, germline mutations in the *RPS20* gene could be identified using only a single four-generation family, which is probably unique since similar mutations have so far not been reported in other families [28].

Another strategy may be a search for founder mutations. The recruitment of families with similar phenotypes from local populations may be of help for such strategies. This approach has e.g. led to the identification of *POT1* mutations in cutaneous melanoma [131] and an *NTHL1* variant in patients with hereditary polyposis by our group [29].

Studies of Mendelian disorders suggest that the patient selection step may be crucial for increasing the power of the studies to reveal rare alleles with a high to moderate penetrance. This is especially so since in a cohort of resembling patients it is more likely that a single gene or a few genes with similar function will underlie a specific phenotype. This, in turn, would be more obvious in a plethora of non-causative rare variants [117–122] than would be the identification of a candidate gene by mere prioritization on an ad hoc hypothesis basis with the exclusion of common variants. Possibly, some CRC risk factors may be too rare (e.g. *RPS20*) or their penetrance may be too low to give rise to a distinctly recognizable phenotype. Nevertheless, the advantages of unravelling Mendelian or near-Mendelian CRC conditions are two-fold. First, they may reveal novel pathways involved in CRC predisposition that can be used in further candidate gene identification approaches. Second, the removal of resolved cases from cohorts will increase the specificity of novel searches for the underlying genes and/or pathways (Fig. 3).

Another step to improve the power of studies is to assemble a carefully selected set of normal controls for comparison. As many Mendelian disorders are rare and highly penetrant, it is common to compare the frequency of variants to population-matched controls from public databases, as exemplified by studies that identified the cancer predisposition genes *MAX* [126] and *POLD1*/*POLE* [27]*.* Given the fact that CRC is a common phenotype, selecting age- and population-matched controls may not be trivial, but unrelated gender- and age-matched family members may serve as proper and easily accessible controls.

#### Candidate gene and variant selection

The bottleneck in the MPS study design is rapidly moving away from the performance of genome-wide screens towards the development of bioinformatic tools for the selection of variants and the introduction of comprehensive pipelines for sequence analysis (see for detailed reviews [132, 133]). Particularly, the variant and gene selection step by which the vast number of identified variants is reduced is important (Fig. 3). The approach chosen for prioritization depends on prior knowledge of the patient selection criteria (as described above), the damaging effect of variants encountered in the genes, the inheritance model and the function of the genes affected [129]. Such approaches are not trivial as they are based on selecting one or two causative variants from several million variants that usually result from genome-wide sequencing efforts [117–122]. Overlap, linkage, trio-based sequencing (i.e., patient and parents) and shared homozygosity can be used to augment the prioritization of variants and genes in the exomes of patients with Mendelian conditions [105, 106]. The application of such strategies is not limited to WES but can also be used for WGS [107, 134].

For oligogenic disorders, detailed knowledge of gene and variant function remains important. A thorough understanding of the underlying biology can be of help to specify pathways and gene functions. Gala and colleagues focused in a recent sessile serrated polyposis study, with an increased CRC risk, on truncating variants in oncogene-induced senescence pathways. In doing so, they found several variants in five genes that were enriched in 20 patient-derived exomes as compared to 4500 exomes from healthy individuals deposited in a public database [135]. Although at least one of these variants (p.E49* in *PIF1*) appears to be more common in the healthy population than originally assumed and, therefore, is less likely to be causative (Weren and Herwaarden, personal communication), the study illustrates how insight into the genetic and functional interplay between different genes can assist in variant prioritization. Obviously, such a selection may also introduce biases since it is limited to known pathways. Therefore, further corroborative evidence is required to establish the causality of particular genes and variants.

#### Corroborative evidence to support causality

In order to establish a causal relationship between a variant and its predisposition to cancer, additional evidence is usually required (Fig. 3). This evidence may include (1) recurrence in cases versus controls, (2) co-segregation in families, (3) second-hit mutations in tumours and (4) modelling functional effects.

*Recurrence in cases* versus *controls* One of the best ways to proof that a rare variant, or a rarely mutated gene, plays a role in a Mendelian disease is by independent replication in a validation cohort. Such a replication is considered to be a strong indication that a candidate variant is not the product of an ascertainment bias [129]. Most recent studies that utilized MPS to identify the cause of Mendelian cancer predisposition syndromes aimed at addressing this point [27, 123, 125–128, 130, 136, 137]. Also, for moderately penetrant variants such a replication is considered to be one of the key requirements to demonstrate that a specific or comparable variant recurrently occurs in other patients. To really proof an association, and to assess the variation in the normal population, screening of a large control cohort is needed, the size of which depends on the size of the effect to be investigated. As discussed below, however, further considerations to lend adequate statistical power to such screens will become increasingly important. In cases where replication turns out to be difficult beyond the initial discovery cohort, additional functional evidence or familial co-segregation may be employed to underscore pathogenicity.

*Co-segregation in families* To further assess the penetrance of a candidate risk allele and to check whether the anticipated inheritance model is correct, an assessment of the presence or absence of the candidate risk allele in respectively affected and unaffected family members should be performed. As most Mendelian cancer predisposition syndromes by definition feature either a dominant or a recessive pattern, this step can be very informative. Co-segregation analyses have, for instance, been used to substantiate the implication of *POLD1*, *POLE* and *NTHL1* in CRC [27, 29]. Although there may be considerable ascertainment biases in the selection of families, and cancer syndromes may not exhibit a complete penetrance due to a multifactorial etiology, a familial risk is expected to contribute to our understanding of CRC development.

Translating this approach to moderately penetrant risk variants will, therefore, be important for an accurate risk assessment. A mutation may be significantly associated with early-onset CRC, but may not per se lead to a strong family history of CRC. This notion may be explained by the ‘genetic background’ in such families. Current study designs are not suited to detect polygenic inheritance in cancer. Indeed, as of today, only a few reports have been published that describe interactions between known predisposition genes, including *MUTYH* and *OGG1* [138], *MSH6* and *APC* [139], and *MSH2* and *APC* [140].

*Second-hit mutations in tumours* One of the earliest forms of evidence used to support the causality of mutations was based on Knudson’s two-hit hypothesis [141]. Indeed, many of the well-established cancer predisposition genes, such as those underlying FAP and Lynch syndrome follow this scenario, as described above. Also, some of the recently discovered cancer predisposing genes appear to act in accordance with this scenario, such as for instance the *MAX* [125], *SMARCE1* [126] and *BAP1* [127] genes. Subsequent elaborate work on this scenario has revealed that (partial) deregulation of tumour suppressor genes and/or proto-oncogenes may add to the risk of tumour formation [142]. Therefore, this scenario should not be regarded as absolute. Perhaps, moderately penetrant risk factors may not exhibit second hits, as their effects are less prominently associated with gene dosage.

Instead of limiting mutation analyses to hits in the second allele of a candidate gene, more insight may be gained from genome-wide profiling through MPS. It is well-established now that specific cancer syndromes may follow distinct mutational pathways as has, for instance, been observed in Lynch syndrome (see above), and *MUTYH*-associated polyposis (MAP) [23]. Also, recent studies on CRC predisposition syndromes such as PPAP [27] and NAP [29] have revealed mutation profiles that are specifically associated with defects in DNA repair pathways. Given that MPS continuously improves the ease at which mutations can be detected, the generation of mutation profiles may become a cost-effective way to phenotype heritable tumours [9, 143, 144]. This concept, also known as endophenotyping, may make a clear difference in the study of CRC predisposition.

*Modelling functional effects* As novel genes and variants continue to be implicated in cancer predisposition, it is becoming increasingly imperative to unravel the functional consequences of such genes and variants. Well-established models, such as mouse models, are available for at least some of the currently known cancer predisposition syndromes. Existing knockout models of the *POLE* and *NTHL1* genes have for instance been used to support their implication in CRC predisposition [27, 29]. In the absence of such in vivo models, in silico models can be used to make an educated guess of the functional consequences of specific variants. Ultimately, proof can be delivered by in vitro modelling. For variants in e.g. DNA repair genes this can be done through their exogenous expression in mammalian or yeast cells and assessment whether the sensitivity to DNA damaging agents has increased, as has been shown for *ERCC4* [128] and *POLE* [27]. Also, the effects of variants on downstream signalling can be investigated. This has e.g. been done to show the effect of *TGFBR1* variants on TGF-β signalling through SMAD2/3 [136]. Another example represents the influence of *RHBDF2* variants on EGFR signalling [145]. In the case of *SASH1* mutations, wound-healing assays on control and patient-derived fibroblasts were performed to reveal alterations in pathways governing cell migration [146]. It is anticipated that functional evidence will play an increasingly important role in CRC research. Indeed, in the recent literature, functional analyses have been used to implicate genes in gastrointestinal predisposition syndromes when other criteria such as replication could not be met due to the rarity of pathogenic mutations in the underlying genes. The implication of *RPS20* in a single family with CRC (see above) was for instance supported by showing a defect in pre-rRNA maturation in patients with mutations in this gene [28]. Also, germline mutations in the *IPMK* gene were found to increase the resistance to apoptosis in cell lines derived from a single family with small intestinal carcinoids [147].

### Beyond Mendelian disease gene identification

As alluded to above, novel insights gained from MPS approaches for Mendelian conditions may be limited in what they can contribute to our understanding of CRC predisposition. There are three possible reasons for this: (i) the underlying risk models may not be applicable to CRC, (ii) a significant part of the heritability may result from non-coding variants and (iii) not all familial CRC cases may result from genetic defects.

Firstly, few moderately penetrant genetic risk variants have been studied so far, and their contribution may deviate from currently known inheritance models. Most of our knowledge of moderately penetrant CRC risk variants stems from candidate gene approaches, of which only a few have significantly contributed to novel insights [64]. Interestingly, risk variants may also act as modifiers, which affect the penetrance, dominance, expressivity and pleiotropy of inherited traits [148]. An early example of a role of modifiers in CRC came from a FAP model in mice: *APC* ^min^ [149]. These mice have an average lifespan of 120 days in which they develop multiple polyps and CRC [149]. Through crossing with another mouse strain, a novel strain was obtained of which the mice lived disease-free for almost 300 days, suggesting that the *APC* ^min^ locus was influenced by a modifier [149]. Subsequently, the genetic locus for this modifier was mapped and named modifier of min (*mom*) [150]. In recent years several additional modifiers have been identified and mapped, indicating that modification is a recurring theme, particularly in the WNT signalling cascade [151–153]. Similar observations have been made in mouse models for DNA repair genes. For instance, in a model reminiscent of heterozygous *BLM* ^Ash^ carriers, in which CRC predisposition is only found in an *APC* ^min^ background [154], the overall predisposition effect depends both on *BLM* ablation and on the mouse strain used [155–157]. Such effects are generally not accounted for in Mendelian disease gene and GWAS studies. As a consequence, care should be taken when applying Mendelian prioritization guidelines to detect moderately penetrant CRC risk variants.

Secondly, the criteria used for Mendelian disease genetics are mostly based on the protein-coding regions of the genome (i.e., the exome). While the human exome itself still seems to be ill-defined [158], another significant part of the heritability may reside outside these regions of the genome. Many of the variants identified by GWAS are in fact located outside coding regions [90]. Also, variants implicated in Mendelian cancer syndromes may not necessarily be coding, examples of which include mutations in the *MLH1* 5’UTR region associated with CRC [159, 160] and presumed enhancers that may affect the expression of *EPCAM*-*MSH2* read-through transcripts associated with CRC in *EPCAM* deletion carriers [161]. Likewise, a 40-kb duplication upstream of *GREM1* has been linked to hereditary mixed polyposis syndrome by effecting an increased and ectopic expression of the BMP antagonist GREM1 [48]. Also primary epimutations, that is aberrations in gene expression due to an altered epigenotype not linked to genomic variation, have been suggested to play a role in cancer predisposition [162]. Examples include imprinting of the *H19* locus in Beckwith–Wiedemann syndrome [163] and the de novo methylation of *MLH1* associated with sporadic Lynch-syndrome [164–166]. It is reasonable to assume that many additional, also moderately penetrant, risk factors reside in non-coding regions. Therefore, care should be taken to not overrate the utility of exomes.

Thirdly, not all CRC cases with a suspected heritability may be caused by genetic variants. Recent work of Tomasetti and Vogelstein suggest that only a few mutations are required for CRC to become manifest [167, 168]. Their proposition raises some concern about how much of the CRC heritability still remains to be discovered. Furthermore, mere chance together with the above-mentioned modifying effects have not been taken into account in current heritability estimates [167, 169, 170]. Therefore, it is difficult to provide an accurate estimate of the remaining heritability of CRC and how much of it may be attributed to which class of variants.

Although the above limitations may appear challenging, some promising strategies and developments have been outlined in the current literature, i.e., (1) rare variant association studies, (2) whole-genome sequencing, (3) high-throughput functional genomics and (4) somatic driver mutation and germline mutation overlap (Fig. 3).

#### Rare variant association studies to power discoveries

Most of the studies presented in the survey above have sought to prioritize variants under the premise that they would be similar to Mendelian conditions. As already pointed out, the insights gained by these approaches have been limited. A particular shortcoming is the strong signal-to-noise ratios in WES and WGS experiments [171]. One way to filter out causative variants from ‘noise’, such as large numbers of neutral variants, may be to enhance the power of associations in large groups of cases and controls. Simulations have indicated that studies in which several thousands of samples are included may be adequately powered to detect loci explaining ~1 % of the phenotypic variance underlying a common dichotomous trait in WES screens [172]. It has been proposed that the power of such approaches could be enhanced by focusing on isolated populations, de novo mutations and/or specific genomic regions [171]. For WGS, probably much more cases and controls are needed, demanding cohort sizes similar or even larger than those used in recent GWAS. As GWAS consortia have already collected such cohorts, these may also be used for WGS. This notion may be a basis for future patient recruitment (Fig. 3).

#### WGS and the elucidation of non-coding and complex variation

From studies comparing WES with WGS, it has been concluded that WGS is much more sensitive than WES in calling single-nucleotide variants and short insertions/deletions (indels) at a comparable coverage [173, 174]. Moreover, it was found that WGS avoids the biases intrinsic to enrichment, thereby delivering improved uniformity of read coverage and reduced bias of allele ratios across the entire genome [173. This also translates into a greater ease in the detection of copy number variation across the genome by WGS [173]. It is considered likely that with further improvements in the technology towards applications of ‘third generation sequencing’, such as the Nanopore [175, 176] or PacBio platforms [177], increasing read lengths will allow the delineation of complex structural rearrangements and the phasing of variants by a single WGS experiment (Fig. 3). Currently, however, our understanding of non-coding variation is still limited. Therefore, a further development of conceptualized data interpretation approaches [178–180], as well as functional genomics, will be required.

#### High-throughput functional genomics

Recently, high-throughput functional technologies have become available to study specific gene functions and to develop human disease models through the application of genomic engineering via nuclease-induced genome modifications. These technologies allow forward as well as reverse genetic screens [181]. In particular, the CRISPR/Cas9 system has become a popular option based on its simplicity and versatility [182]. Findlay and colleagues already used the CRISPR/Cas9 system to e.g. mutagenize a region of 6 nucleotides in exon 18 of the *BRCA1* gene with all possible hexamers and compared their impact on nonsense-mediated decay and exonic splicing [183].

Besides the recapitulation of specific variants, functional genetic analyses have become feasible in a much broader context in both in vitro and in vivo models. Promising models include organoids, which represent in vitro culture systems to grow cells in a three-dimensional (3D) organ-like fashion, such as intestinal epithelial structures derived from Lgr5-positive colonic crypt stem cells [184]. Importantly, these organoids are amenable to high-throughput genetic manipulation. Recently, the utility of these organoids was demonstrated by introducing mutations in the key driver tumour suppressor genes *APC*, *SMAD4* and *TP53*, and the oncogenes *KRAS* and *PIK3CA*, in colonic organoids via a knock-out/knock-in approach to mimic tumorigenesis according to the classical adenoma-carcinoma sequence model [185, 186]. When comparing the tumorigenic and metastatic properties of these organoids, it was found that ‘driver’ pathway mutations are sufficient for clonal expansion and features of invasive carcinomas, but that additional molecular lesions are required for invasive behaviour [185, 186]. These findings underscore the notion that knowledge on genetic background and environmental variation may be important for our understanding of CRC development. As such, recent efforts to build organoid biobanks from CRC patients may hold promise for the elucidation of individual genetic and environmental risk factors [187] in a high-throughput setting. Last but not least, the findings may be translated into animal models and, ultimately, humans.

#### Using somatic driver mutations to identify novel predisposition genes

An interesting feature of many of the CRC predisposing variants is the apparent overlap between the corresponding pathways that drive cancer development somatically and those that predispose genetically. This notion is not new, as the very first models of cancer development such as the Knudson’s two-hit hypothesis discussed above originated from these parallels. Recent large-scale profiling efforts of cancer genomes have revealed genes frequently affected by somatic mutations in CRC [9, 143, 144]. These efforts were motivated by the aim to identify driver mutations. In a recent review, a comparison was made between drivers in the COSMIC (Catalogue of Somatic Mutations in Cancer) database featuring 468 genes that are somatically mutated in cancers, and known germline predisposition genes [188]. The result was that ~10 % of somatically mutated cancer genes also confer susceptibility to cancer when present as mutant variants in the germline, but that 40 % of the germline-mutated predisposition genes can also be mutated somatically in tumours [188]. The author suggested that this discrepancy may at least in part represent a bias due to the scarcity of studies on germline-associated mutations in tumours [188]. Indeed, holistic studies of somatic and germline variants in tumour material to reveal oncogenic drivers are getting increasingly popular [189]. Most of the current studies are limited because of their small numbers and the resulting lack of power. Therefore, more systematic screens of tumour and matched germline material using large patient cohorts will be needed. Since the screening for somatic mutations in cancer cells has been among the first fields to harness the power of MPS, and especially WGS [190], the methods used to identify driver mutations are better established than those to identify germline variants. Consequently, although missing driver variants have been reported, the identification of driver variants in (non-)coding regions [191] may lead to the discovery and improved prioritization of germline variants (Fig. 3).

## Conclusions

With the advent of MPS, expectations have been high that this technology may be able to reveal much of the missing heritability of CRC. Next to identifying a number of strong candidate genes affected by clearly interpretable rare risk variants, the application of MPS to CRC has thus far led to a plethora of potential candidate genes whose role still remains to be established. In this review, we have outlined how further adjustments of experimental approaches and strategies may be of help to obtain final evidence on true CRC risk factors as also on their clinical relevance. Knowledge on functional networks of known (highly penetrant) genetic risk factors may be rewarding in this respect, as they may reveal novel pathways involved in cancer predisposition. In the end, it is anticipated that the availability of large multi-center datasets from clinically well-defined patients and controls will yield enough power to perform independent comprehensive meta-analyses, which may unravel the complex interplay between common and rare variants within genes or intergenic regions, and to make an accurate individualized estimate for the risk to develop CRC (and other clinical entities).
